# Mechanical Simulation of Thermoplastic Composite Fiber Variable-Angle Laminates

**DOI:** 10.3390/ma13153374

**Published:** 2020-07-30

**Authors:** Zhongliang Cao, Dengke Guo, Hongya Fu, Zhenyu Han

**Affiliations:** 1 School of Mechanical Engineering, Jiangsu Institute of Technology, Changzhou 213001, China; 2 School of Mechatronics Engineering, Qiqihar University, Qiqihar 161001, China; gdkwawj@outlook.com; 3 School of Mechatronics Engineering, Harbin Institute of Technology, Harbin 150001, China; hongyafu@hit.edu.cn (H.F.); hanzy@hit.edu.cn (Z.H.)

**Keywords:** thermoplastic fiber, automatic placement, variable-angle trajectory planning, mechanical properties

## Abstract

By changing the placement angle of the placement path, the fiber direction can be controlled and adjusted to change the load distribution in the laminate, and the stress and natural frequency performances of the laminate can then be altered to finally obtain laminates with desired mechanical properties. In this paper, the finite element analysis model of variable-stiffness laminates is established based on the fiber placement reference path defined by the Bezier curve method. Based on the analysis of the mechanical properties of the thermoplastic fiber variable-angle laminates obtained by variable-angle trajectory planning, the changes in the stress and deformation of the thermoplastic fiber variable-angle laminate with the connection point parameter β under a compressive load are analyzed. The influence of the parameter β on the static performances of the variable-angle laminates is studied. The simulation results indicate that the maximum stress of the laminate increases first and then decreases as the parameter β increases and reaches the maximum value when the parameter β is 0.5. The minimum stress also shows the same trend as that of the maximum stress and reaches the minimum value when the connection point parameter β is 0.3. The deformation of the variable-angle laminates varies with the change of the connection point parameter β. The maximum deformation increases at first and then decreases for the laminate with the parameter β increasing and reaches the maximum value when the parameter β is 0.8. The minimum deformation of the laminate decreases initially and then increases as the connection point parameter β increases and reaches the minimum value when the parameter β is 0.6. The deformation gradually decreases from the upper and lower ends to the middle, and the deformation area has a symmetrical form. The initial regular rectangular area gradually changes to an elliptical distribution and the area of maximum deformation gradually decreases.

## 1. Introduction

**Figure 1:**
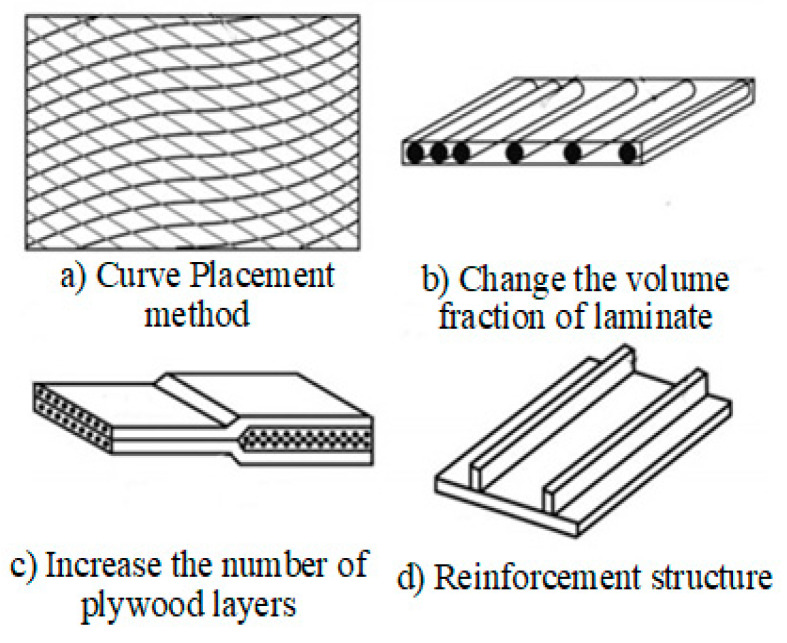
Four forms employed to achieve variable-stiffness laminates. (**a**) Cure placement method; (**b**) Change the volume fraction of laminate; (**c**) Increase the number of plywood layers; (**d**) Reinforcement structure.

Composite laminates with excellent structural properties have been widely used in the fields of aerospace, bridge construction, wind power, etc. [1]. For composite laminates, the stability problem is often encountered in the study of laminates. At present, constant-angle laminates formed by composite material placement are widely used in aerospace and related fields [2,3,4]. The constant angles of the composite layer are mainly 0°, ±45°, and 90°, and the relevant layers are set according to the mechanical and technological requirements. However, the mechanical properties of composite laminates are greatly limited due to the constant angle of fiber placement. Variable-angle placement can make the fiber trajectory continuously change in the same plane in real time. Laminates with continuously changing fiber placement angles are called “variable-angle laminates”, which can be controlled by changing the design parameters of the placement trajectory [5,6]. By changing the design parameters of the placement trajectory, the fiber direction can be controlled and adjusted to change the load distribution in the plane, and the stress and natural frequency characteristics are changed. Finally, the ideal mechanical properties of laminates are obtained. Compared with thermoset composite materials, thermoplastic composite materials have broad application prospects with a good weldability, impact toughness, recyclability, chemical corrosion resistance, and other advantages [7,8,9]. Because of the application advantages, thermoplastic composite materials have become the research focus in the field of the development of composite materials. In recent years, thermoplastic fiber variable-angle placement laminates have gradually been applied in various practical engineering fields [10]. At present, there are four main design forms for achieving composite material variable-stiffness laminates, as shown in Figure 1.

When using the variable-angle trajectory application in actual placement technology, the placement structure of composite laminates gradually changes from a constant angle to a variable angle, hence the named variable-angle ply [11]. The advantage of variable-angle composite laminates is that it can improve the mechanical properties of the whole laminate and reduce or avoid the stress concentration problem by changing the fiber trajectories and keeping the thickness of the laminate unchanged [12,13,14]. In the 1990s, Gürdal et al. changed the fiber placement angle along the reference geometric axis to form a curved fiber path, and the concept of variable-stiffness laminates was introduced. The effect is obviously improved compared with the straight-line placement method [15,16]. From the literature published in recent years, there have been many analyses on the buckling performance and other mechanical properties of laminates constructed by a linear variable-angle trajectory, such as those by Marouene, Sabido, Milazzo, Madeoaand, and other researchers [17,18,19,20]. Alhajahmad et al. [21] used Lobatto–Legendre polynomials to describe the reference path and used the finite element method and Ritz method to analyze the buckling and failure forms of variable-stiffness laminates at the same time. Lopes et al. [22] studied the design freedom brought by the variable-stiffness layer to the laminates and optimized the maximum buckling load. Surya et al. [23] discussed the dynamic instability of variable-angle laminates. Kazem et al. [24] proposed a new method called the “defect layer” method and found that the influence of gaps and overlapping on the compressive strength depended on the location of the defect and the percentage of defect area. This method has a great effect on the stiffness and buckling load of laminates. Ribeiro et al. [25] summarized the current design methods and related design forms of variable-angle laminates, and discussed some of the problems with variable-angle laminates. The research results above show that variable-angle placement can change the buckling performance, but these studies mostly focused on the linear variable-angle method for carrying out buckling and failure analyses. At present, there is no research on the static mechanical properties of the Bezier curve method, especially on the relationship between the design parameters and the mechanical properties in the variable-angle trajectory model.

In this paper, variable-angle laminates based on the quadratic Bezier curve method are studied in terms of the stress and deformation of thermoplastic variable-angle laminates, and ABAQUS is used for simulation calculation. A static analysis of the variable-angle laminates is employed to study the influence of the connection point parameter β on the static characteristics of the variable-angle laminate in-plane stress distribution and deformation under the action of compressive load.

## 2. Mathematical Model of Variable-Angle Trajectory Planning Based on the Quadratic Bezier Curve Method 

**Figure 2:**
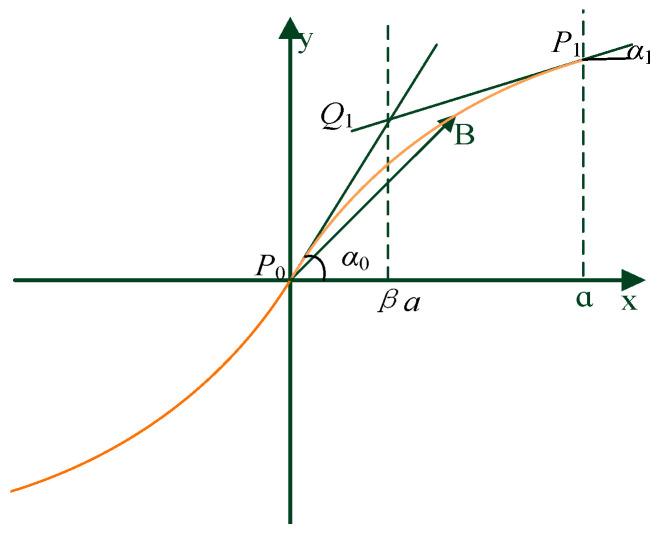
Quadratic Bezier curve.

The primary task of variable-angle trajectory planning is to establish a reference trajectory. The establishment of the reference trajectory plays an important role in thermoplastic fiber placement. The quality of the reference trajectory will directly affect the quality and mechanical properties of the laid product. At present, most of the research on reference trajectories is based on the linear variable-angle method, and research results on nonlinear change of the placement angle in fiber trajectories are scarce. In the automatic placement process of thermoplastic fibers, variable-angle trajectories are generally defined based on mathematical equations or formulas. In order to expand the design scope and design freedom of variable-angle trajectories and improve the designability of variable-angle trajectories, this paper uses the Bezier curve method to construct a variable-angle reference trajectory and mathematical model. The reference trajectory constructed by the quadratic Bezier curve is illustrated in Figure 2. It can be found from the figure that *P*_0_ is the starting point of the curve; *P*_1_ is the end point; and the corresponding tangent angles are *α*_0_ and *α*_1_, respectively. Meanwhile, the tangent lines of the starting point and the end point intersect at any point *Q*_1_. *β* is defined as the connection point parameter and determines the position of *Q*_1_. The variation law of the slope angle of the whole curve can be changed by *β*. In this paper, the range of *β* is [0, 1].

The equation of the quadratic Bezier curve can be deduced by the relevant parameters of each point in Figure 2.

(1) $$\left\{ \begin{array}{l} \vec{B}\left(t\right)=\left(1-t^{2}\right)\vec{P_{0}}+2\left(1-t\right)t\vec{P_{0}Q_{1}}+t^{2}\vec{P_{0}P_{1}},t\in \left[0,1\right]\\ \vec{P_{0}}=\left(0,0\right)\\ \vec{P_{0}Q_{1}}=\left(\beta a,\beta a\tan\alpha_{0}\right)\\ \vec{P_{0}P_{1}}=\left(a,\beta a\tan\alpha_{0}+\left(1-\beta \right)a\tan\alpha_{1}\right) \end{array}\right.$$

It can be seen from the figure that the location of the connection point *Q*_1_ is between the starting point *P*_0_ and the end point *P*_1_. The position of *Q*_1_ will vary with the change of the connection point parameter *β*. The position of *Q*_1_ will approach the end point *P*_1_ with the increase of *β*. Therefore, the *x* and *y* coordinates of any point on the quadratic Bezier curve can be expressed as

(2) $$x=a\left(\left(1-2\beta \right)t^{2}+2\beta t\right),0\leq x,t\leq 1$$

(3) $$y=a\left(\left(\left(1-\beta \right)\tan\alpha_{1}-\beta \tan\alpha_{0}\right)t^{2}+2\beta \tan\alpha_{0}t\right)$$

According to the trigonometric relationship, the tangent angle and variable relation of any point on the quadratic Bezier curve can be obtained in the following Equations (4) and (5):

(4) $$\theta =\tan^{-1}\left(\frac{dy_{1}}{dt}\frac{dt}{dx_{1}}\right)=\tan^{-1}\left(\frac{t\left(1-\beta \right)\tan\alpha_{1}+\left(1-t\right)\beta \tan\alpha_{0}}{\left(1-2\beta \right)t+\beta }\right)$$

(5) $$t=\left\{ \begin{array}{ll} x & \beta =0.5\;\\ \frac{-\beta +\sqrt{\beta^{2}+\left(1-2\beta \right)x_{1}}}{\left(1-2\beta \right)} & \beta \neq 0.5 \end{array}\right.$$

Because the connection point parameter *β* changes, the corresponding angle trajectory also changes so that multiple initial reference trajectories can be realized, and the design degree of freedom becomes greater in the angle change process. More details about the construction method and changes with connection point parameters of the Bezier curve can be found in reference [26].

## 3. Finite Element Analysis Model of the Variable-Angle Layer

**Figure 3:**
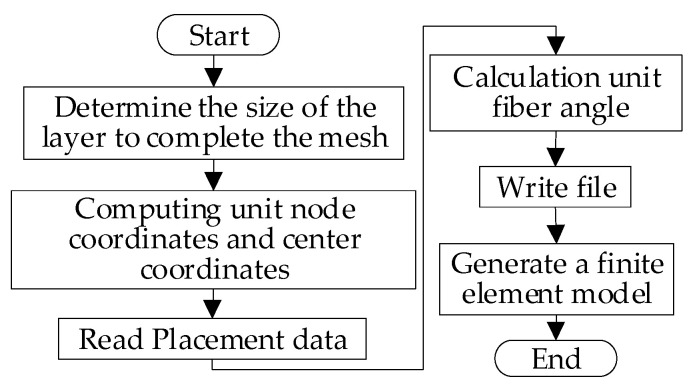
Process of creating an analytical model for variable-angle laminates.

**Figure 4:**
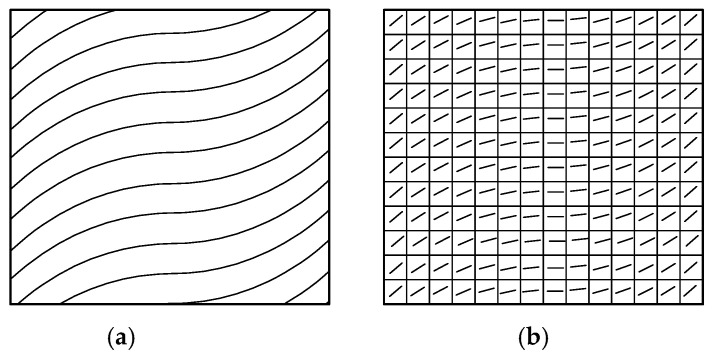
Schematic of the mesh after the variation angle layer has been divided: (**a**) Variable-angle layer, and (**b**) schematic diagram obtained after meshing.

The placement angle in the variable-angle reference trajectory changes with the change of the trajectory, which will lead to a great change in the mechanical properties of the fiber. The mechanical properties of variable-angle laminates will change with the change of the placement angle of the fiber. At present, existing commercial finite element analysis software, such as ANSYS (Ansys12.0, Ansys, Inc., Canonsburg, PA, USA) and ABAQUS (Abaqus 6.1, Dassault Simulia, Inc., Waltham, ME, USA), can only carry out a mechanical analysis for constant-angle laminates, and a corresponding mechanical performance analysis cannot be performed for variable-angle laminates. In this paper, the secondary development form of MATLAB (Matlab 7.10, MathWorks, Inc, Natick, MA, USA) and ABAQUS (Abaqus 6.1, Dassault Simulia, Inc., Waltham, ME, USA) is used, and the analysis model of variable-angle laminates is established by writing the corresponding program to define the properties and fiber angle. With reference to the meshing tool in the finite element software, the variable-angle layer is divided into a certain number of mesh units. When the number of mesh units is sufficient, the fiber angle presented in each mesh unit can be approximately regarded as unchanged. It is processed according to the fixed-angle laminate board unit to generate a read file of the variable-angle laminate board and finally imported into ABAQUS for corresponding mechanical performance analysis. Therefore, all results are achieved from FEM solutions. The process of creating an analysis model for a variable-angle laminate is illustrated in Figure 3 and Figure 4 is a schematic diagram of the mesh after the variable angle has been divided. Figure 4a shows the form of each track, while Figure 4b is a schematic diagram obtained after meshing. As can be seen from the figure, the fiber angle in each mesh is a fixed value.

**Figure 5:**
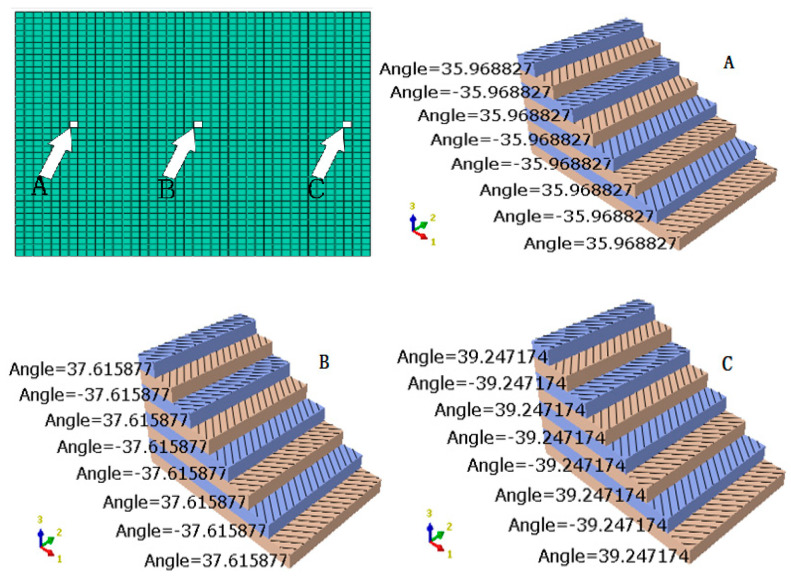
Schematic of the element angle in a variable-angle laminate. (**A**) Arbitrary unit A; (**B**) Arbitrary unit B; (**C**) Arbitrary unit C.

The analysis model [0 ± < 20 (0.6) 60) >]_2s_ of the layer is imported into ABAQUS. The unit angle in variable-angle ply is queried by using the software query function. Figure 5 presents a unit angle schematic diagram of the variable-angle layer. The first picture on the upper left is a variable-angle composite laminate after meshing. Three units can be selected at different positions in the laminate. From the three positions of A, B, and C, it can be seen that the angle changes between the different position grids in the same layer. The angles of two adjacent layers are equal, but the signs are opposite. The finite element model can well-reflect the fiber placement angle change. At the same time, it can also be concluded that the denser the mesh is divided, the more continuous the angular trajectory is, which is close to the actual variable-angle placement structure. In this paper, it is assumed that the interlaminars are completely fused, with no manufacturing defects in the analysis model.

At the same time, the internal force obtained by the model [0 ± < 45 (β = 0.6) 75 >]_9s_ is compared with that of the model [0 ± < 45|75 >]_9s_ mentioned in reference [27] to verify the validity of the modeling method presented in this paper. It can be seen from the comparison that the distribution trends of the internal force of both models display great consistency. When β equals 1 or 0, the trajectories of these two types are expressed as constant-angle trajectories. Constant-stiffness laminates [0 ± < 20 (1) 60 >]_2s_ can be obtained by setting β to 1. The theoretical buckling load is calculated and compared with that solved by ABAQUS. The relative error of the buckling load between the theoretical method and the FEM method is about 2%, which indicates that the established model based on the Bezier curve method is accurate for analyzing the variable-angle laminates.

## 4. Study on Statics of Thermoplastic Fiber Variable-Angle Laminates

### 4.1. Constitutive Equation of Variable-Angle Laminates

**Figure 6:**
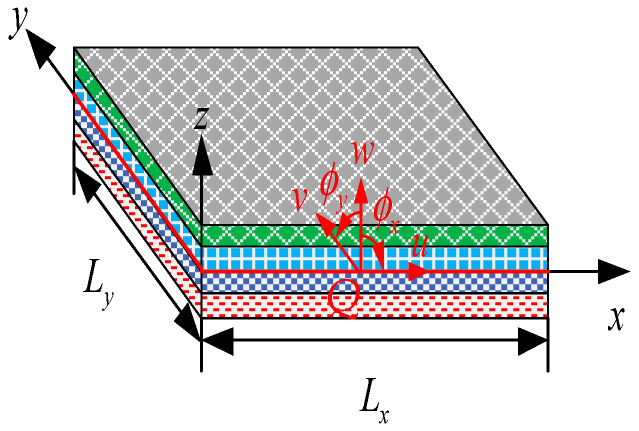
Schematic diagram of the element coordinates.

For variable-angle laminates, the stress in the laminate will change as the fiber placement angle changes. Figure 6 is a schematic diagram of the coordinate system of elements. For laminate elements, according to the first-order shear theory, it can be assumed that the normal of the middle plane is kept as a straight line, but not perpendicular to the middle plane [28]. Then, the displacement field at any point (*u*, *v*, *w*) in the laminate can be expressed as

(6) $$u(x,y,z,t)=u_{0}(x,y,t)+z\phi_{x}(x,y,t)$$

(7) $$v(x,y,z)=v_{0}(x,y,t)+z\phi_{y}(x,y,t)$$

(8) $$w(x,y,z,t)=w_{0}(x,y,t)$$

where *u*_0_, *v*_0_, and *w*_0_ are the translational displacements of corresponding point *Q* along the *x*, *y*, and *z* coordinate directions, respectively; *ϕ*_x_ and *ϕ*_y_ are the angular displacements of the normal of the middle plane with respect to *y* and *x* coordinates, respectively; and *L_x_* and *L_y_* are the length along the *x* and *y* direction, respectively. Based on the small deformation assumption, the linear strain of the S4R element can be obtained as

(9) $$\varepsilon_{xx}=\varepsilon_{xx}^{\left(0\right)}+z\varepsilon_{xx}^{\left(1\right)},\;\varepsilon_{yy}=\varepsilon_{yy}^{\left(0\right)}+z\varepsilon_{yy}^{\left(1\right)},\;\gamma_{xy}=\gamma_{xy}^{\left(0\right)}+z\gamma_{xy}^{\left(1\right)},\;\gamma_{xz}=\gamma_{xz}^{\left(0\right)},\;\gamma_{yz}=\gamma_{yz}^{\left(0\right)}$$

The midplane strain $\varepsilon_{xx}^{\left(0\right)}$, $\varepsilon_{yy}^{\left(0\right)}$, $\gamma_{xy}^{\left(0\right)}$, $\gamma_{xz}^{\left(0\right)}$, $\gamma_{yz}^{\left(0\right)}$, bending deflection $\varepsilon_{xx}^{\left(1\right)}$, $\varepsilon_{yy}^{\left(1\right)}$ and twist rate $\gamma_{xy}^{\left(1\right)}$ can be respectively expressed as

$$\varepsilon_{xx}^{\left(0\right)}=\frac{\partial u_{0}}{\partial x},\;\varepsilon_{yy}^{\left(0\right)}=\frac{\partial v_{0}}{\partial y},\;\gamma_{xy}^{\left(0\right)}=\frac{\partial v_{0}}{\partial x}+\frac{\partial u_{0}}{\partial y}$$

(10) $$\gamma_{xz}^{\left(0\right)}=\frac{\partial w_{0}}{\partial x}+\phi_{x},\;\gamma_{yz}^{\left(0\right)}=\frac{\partial w_{0}}{\partial y}+\phi_{y}$$

$$\varepsilon_{xx}^{\left(1\right)}=\frac{\partial \phi_{x}}{\partial x},\;\varepsilon_{yy}^{\left(1\right)}=\frac{\partial \phi_{y}}{\partial y},\;\gamma_{xy}^{\left(1\right)}=\frac{\partial \phi_{y}}{\partial x}+\frac{\partial \phi_{x}}{\partial y}$$

Each plate element can be equivalent to a composite laminate composed of N equal-thickness layers. Each layer is composed of fibers that are parallel to each other and made of the same continuous orthotropic material, and are embedded in the matrix material. The main function of the matrix material is to connect these fibers together to transfer the shear stress between the fibers. According to the macro mechanics theory, the following assumptions can be made for the composite laminate:

(1) In each layer of the composite laminate, the fiber is always parallel to the top and bottom of the layer;

(2) The angle between the fiber in each layer and that in other layers remains constant.

At the micro level, according to Hooke’s law of orthotropic materials, the stress–strain relationship of each layer in the material coordinate system (o-*x*_1_*x*_2_*x*_3_) is as follows:

(11) $$\left\{\begin{array}{c} \sigma_{11}^{\left(k\right)}\\ \sigma_{22}^{\left(k\right)}\\ \tau_{23}^{\left(k\right)}\\ \tau_{13}^{\left(k\right)}\\ \tau_{12}^{\left(k\right)} \end{array}\right\}=\left[\begin{array}{ccccc} Q_{11}^{\left(k\right)} & Q_{12}^{\left(k\right)} & 0 & 0 & 0\\ Q_{12}^{\left(k\right)} & Q_{22}^{\left(k\right)} & 0 & 0 & 0\\ 0 & 0 & Q_{44}^{\left(k\right)} & 0 & 0\\ 0 & 0 & 0 & Q_{55}^{\left(k\right)} & 0\\ 0 & 0 & 0 & 0 & Q_{66}^{\left(k\right)} \end{array}\right]\left\{\begin{array}{c} \varepsilon_{11}^{\left(k\right)}\\ \varepsilon_{22}^{\left(k\right)}\\ \gamma_{23}^{\left(k\right)}\\ \gamma_{13}^{\left(k\right)}\\ \gamma_{12}^{\left(k\right)} \end{array}\right\}$$

where $Q_{ij}^{\left(k\right)}$ (*i, j* = 1, 2, ∙∙∙, 6) represents the stiffness parameters that can be defined by the elastic constants of orthotropic materials as follows:

(12) $$Q_{11}^{\left(k\right)}=\frac{E_{1}^{\left(k\right)}}{1-v_{12}^{\left(k\right)}v_{21}^{\left(k\right)}},\;Q_{12}^{\left(k\right)}=\frac{v_{12}^{\left(k\right)}E_{1}^{\left(k\right)}}{1-v_{12}^{\left(k\right)}v_{21}^{\left(k\right)}},\;Q_{22}^{\left(k\right)}=\frac{E_{2}^{\left(k\right)}}{1-v_{12}^{\left(k\right)}v_{21}^{\left(k\right)}},Q_{44}^{\left(k\right)}=G_{23}^{\left(k\right)},\;Q_{55}^{\left(k\right)}=G_{13}^{\left(k\right)},\;Q_{66}^{\left(k\right)}=G_{12}^{\left(k\right)}$$

The constitutive Equation (11) is established at the material coordinates of each layer, but the analysis of the composite laminate structure occurs in the element coordinate system (*x*, *y*, *z*). The fiber angles in the layers contained in the laminated structure are often different, and the material coordinates are not all parallel. Therefore, it is necessary to establish a transformation relationship to transform the stress and strain of each layer into the element coordinate system, so as to obtain a constitutive equation that can be described under the macro mechanics theory. According to the coordinate transformation, the following result is obtained:

(13) $$\left\{\begin{array}{c} x\\ y\\ z \end{array}\right\}=\left[\begin{array}{ccc} m & -n & 0\\ n & m & 0\\ 0 & 0 & n \end{array}\right]\left\{\begin{array}{c} x_{1}\\ x_{2}\\ x_{3} \end{array}\right\}$$

where $m=\cos\theta^{\left(k\right)}$,$n=\sin\theta^{\left(k\right)}$, and $\theta^{\left(k\right)}$ represent the fiber placement angle for each layer. Thereby, both stress and strain are second-order tensors, and the tensor calculation formula is as follows:

(14) $$\left\{\begin{array}{c} \sigma_{xx}^{\left(k\right)}\\ \sigma_{yy}^{\left(k\right)}\\ \sigma_{zz}^{\left(k\right)}\\ \tau_{yz}^{\left(k\right)}\\ \tau_{xz}^{\left(k\right)}\\ \tau_{xy}^{\left(k\right)} \end{array}\right\}=\left[\begin{array}{cccccc} m^{2} & n^{2} & 0 & 0 & 0 & -2mn\\ n^{2} & m^{2} & 0 & 0 & 0 & 2mn\\ 0 & 0 & 1 & 0 & 0 & 0\\ 0 & 0 & 0 & m & n & 0\\ 0 & 0 & 0 & -n & m & 0\\ mn & -mn & 0 & 0 & 0 & m^{2}-n^{2} \end{array}\right]\left\{\begin{array}{c} \sigma_{1}^{\left(k\right)}\\ \sigma_{2}^{\left(k\right)}\\ \sigma_{3}^{\left(k\right)}\\ \tau_{23}^{\left(k\right)}\\ \tau_{13}^{\left(k\right)}\\ \tau_{12}^{\left(k\right)} \end{array}\right\}$$

(15) $$\left\{\begin{array}{c} \varepsilon_{xx}^{\left(k\right)}\\ \varepsilon_{yy}^{\left(k\right)}\\ \varepsilon_{zz}^{\left(k\right)}\\ \gamma_{yz}^{\left(k\right)}\\ \gamma_{xz}^{\left(k\right)}\\ \gamma_{xy}^{\left(k\right)} \end{array}\right\}=\left[\begin{array}{cccccc} m^{2} & n^{2} & 0 & 0 & 0 & -2mn\\ n^{2} & m^{2} & 0 & 0 & 0 & 2mn\\ 0 & 0 & 1 & 0 & 0 & 0\\ 0 & 0 & 0 & m & n & 0\\ 0 & 0 & 0 & -n & m & 0\\ mn & -mn & 0 & 0 & 0 & m^{2}-n^{2} \end{array}\right]\left\{\begin{array}{c} \varepsilon_{1}^{\left(k\right)}\\ \varepsilon_{2}^{\left(k\right)}\\ \varepsilon_{3}^{\left(k\right)}\\ \gamma_{23}^{\left(k\right)}\\ \gamma_{13}^{\left(k\right)}\\ \gamma_{12}^{\left(k\right)} \end{array}\right\}$$

By substituting Equations (14) and (15) into Equation (11), the conversion constitutive equation of the k-th layer can be obtained:

(16) $$\left\{\begin{array}{c} \sigma_{xx}^{\left(k\right)}\\ \sigma_{yy}^{\left(k\right)}\\ \tau_{yz}^{\left(k\right)}\\ \tau_{xz}^{\left(k\right)}\\ \tau_{xy}^{\left(k\right)} \end{array}\right\}=\left[\begin{array}{ccccc} {\bar{Q}}_{11}^{\left(k\right)} & {\bar{Q}}_{12}^{\left(k\right)} & 0 & 0 & {\bar{Q}}_{16}^{\left(k\right)}\\ {\bar{Q}}_{12}^{\left(k\right)} & {\bar{Q}}_{22}^{\left(k\right)} & 0 & 0 & {\bar{Q}}_{26}^{\left(k\right)}\\ 0 & 0 & {\bar{Q}}_{44}^{\left(k\right)} & {\bar{Q}}_{45}^{\left(k\right)} & 0\\ 0 & 0 & {\bar{Q}}_{45}^{\left(k\right)} & {\bar{Q}}_{55}^{\left(k\right)} & 0\\ {\bar{Q}}_{16}^{\left(k\right)} & {\bar{Q}}_{26}^{\left(k\right)} & 0 & 0 & {\bar{Q}}_{66}^{\left(k\right)} \end{array}\right]\left\{\begin{array}{c} \varepsilon_{xx}^{\left(k\right)}\\ \varepsilon_{yy}^{\left(k\right)}\\ \gamma_{yz}^{\left(k\right)}\\ \gamma_{xz}^{\left(k\right)}\\ \gamma_{xy}^{\left(k\right)} \end{array}\right\}$$

where ${\bar{Q}}_{ij}^{\left(k\right)}$ (*i, j* = 1, 2, ∙∙∙, 6) is the conversion of stiffness parameters and can be calculated as

(17) $$\left\{ \begin{array}{l} {\bar{Q}}_{11}^{\left(k\right)}=Q_{11}^{\left(k\right)}m^{4}+2(Q_{12}^{\left(k\right)}+2Q_{66}^{\left(k\right)})m^{2}n^{2}+Q_{22}^{\left(k\right)}m^{4}\\ {\bar{Q}}_{12}^{\left(k\right)}=(Q_{11}^{\left(k\right)}+Q_{22}^{\left(k\right)}-4Q_{66}^{\left(k\right)})m^{2}n^{2}+Q_{12}^{\left(k\right)}(m^{4}+n^{4})\\ {\bar{Q}}_{22}^{\left(k\right)}=Q_{11}^{k}n^{4}+2(Q_{12}^{\left(k\right)}+2Q_{66}^{\left(k\right)})m^{2}n^{2}+Q_{22}^{\left(k\right)}m^{4}\\ {\bar{Q}}_{16}^{\left(k\right)}=(Q_{11}^{\left(k\right)}-Q_{12}^{\left(k\right)}-2Q_{66}^{\left(k\right)})m^{3}n+(Q_{12}^{\left(k\right)}-Q_{22}^{\left(k\right)}+2Q_{66}^{\left(k\right)})mn^{3}\\ {\bar{Q}}_{66}^{\left(k\right)}=(Q_{11}^{\left(k\right)}+Q_{22}^{\left(k\right)}-2Q_{12}^{\left(k\right)}-2Q_{66}^{\left(k\right)})m^{2}n^{2}+Q_{66}^{\left(k\right)}(m^{4}+n^{4})\\ {\bar{Q}}_{44}^{\left(k\right)}=Q_{44}^{\left(k\right)}m^{2}+Q_{55}^{\left(k\right)}n^{2}\\ {\bar{Q}}_{45}^{\left(k\right)}=(Q_{55}^{\left(k\right)}-Q_{44}^{\left(k\right)})mn\\ {\bar{Q}}_{55}^{\left(k\right)}=Q_{55}^{\left(k\right)}m^{2}+Q_{44}^{\left(k\right)}n^{2} \end{array}\right.$$

*N_xx_*, *N_yy_*, and *N_xy_* are the units in plane force on the element cross section; *M_xx_*, *M_yy_*, and *Mxy* are the bending and torque on the element cross section; and *Q_x_* and *Q_y_* are the transverse shear force on the element cross section. According to the laminate theory, the internal force and bending torque are defined as the integral of shell stress in the thickness direction [29]:

(18) $$\begin{array}{l} \left\{\begin{array}{c} N_{xx}\\ N_{yy}\\ N_{xy} \end{array}\right\}=\sum_{k=1}^{N}\int_{z_{k}}^{z_{k+1}}\left\{\begin{array}{c} \sigma_{xx}^{\left(k\right)}\\ \sigma_{yy}^{\left(k\right)}\\ \tau_{xy}^{\left(k\right)} \end{array}\right\}dz\\ \left\{\begin{array}{c} M_{xx}\\ M_{yy}\\ M_{xy} \end{array}\right\}=\sum_{k=1}^{N}\int_{z_{k}}^{z_{k+1}}\left\{\begin{array}{c} \sigma_{xx}^{\left(k\right)}\\ \sigma_{yy}^{\left(k\right)}\\ \tau_{xy}^{\left(k\right)} \end{array}\right\}zdz\\ \left\{\begin{array}{c} Q_{y}\\ Q_{x} \end{array}\right\}=\sum_{k=1}^{N}\int_{z_{k}}^{z_{k+1}}\left\{\begin{array}{c} \tau_{yz}^{\left(k\right)}\\ \tau_{xz}^{\left(k\right)} \end{array}\right\}dz \end{array}$$

By substituting Equation (16) into the above equation, the constitutive relation is obtained as follows:

(19) $$\left\{\begin{array}{c} N_{xx}\\ N_{yy}\\ N_{xy}\\ M_{xx}\\ M_{yy}\\ M_{xy} \end{array}\right\}=\left[\begin{array}{cccccc} A_{11} & A_{12} & A_{16} & B_{11} & B_{12} & B_{16}\\ A_{12} & A_{22} & A_{26} & B_{12} & B_{22} & B_{26}\\ A_{16} & A_{26} & A_{66} & B_{16} & B_{26} & B_{66}\\ B_{11} & B_{12} & B_{16} & D_{11} & D_{12} & D_{16}\\ B_{12} & B_{22} & B_{26} & D_{12} & D_{22} & D_{26}\\ B_{16} & B_{26} & B_{66} & D_{16} & D_{26} & D_{66} \end{array}\right]\left\{\begin{array}{c} \varepsilon_{xx}^{\left(0\right)}\\ \varepsilon_{yy}^{\left(0\right)}\\ \gamma_{xy}^{\left(0\right)}\\ \varepsilon_{xx}^{\left(1\right)}\\ \varepsilon_{yy}^{\left(1\right)}\\ \gamma_{xy}^{\left(1\right)} \end{array}\right\}$$

(20) $$\left\{\begin{array}{c} Q_{y}\\ Q_{x} \end{array}\right\}=\kappa \left[\begin{array}{l} A_{44}A_{45}\\ A_{45}A_{55} \end{array}\right]\left\{\begin{array}{c} \gamma_{yz}^{\left(0\right)}\\ \gamma_{xz}^{\left(0\right)} \end{array}\right\}$$

where *A_ij_* is the tension compression stiffness coefficient; *B_ij_* is the tension compression-bending coupling stiffness coefficient; *D_ij_* (*i, j* = 1, 2, 6) is the bending stiffness coefficient, as follows:

(21) $$\left\{ \begin{array}{l} \{A_{ij},B_{ij},D_{ij}\}=\sum_{k=1}^{N}\int_{z_{k}}^{z_{k+1}}{\bar{Q}}_{ij}^{\left(k\right)}\{1,z,z^{2}\}dz\\ \{H_{44},H_{45},H_{55}\}=\int_{-h/2}^{h/2}\left({\bar{Q}}_{44},{\bar{Q}}_{45},{\bar{Q}}_{55},\right)dz=\sum_{k=1}^{N}\int_{z_{k}}^{z_{k+1}}\left({\bar{Q}}_{44}^{\left(k\right)},{\bar{Q}}_{45}^{\left(k\right)},{\bar{Q}}_{5}^{\left(k\right)}\right)dz \end{array}\right.$$

From the above theoretical derivation, it can be seen that for composite variable-angle laminates, the tension compression stiffness, the tension compression-bending coupling stiffness, and the bending stiffness are all related to ${\overset{-}{Q}}_{ij}$, or, in other words, the fiber placement angle $\theta^{\left(k\right)}$. The constitutive relationship will also change with the change position, resulting in the internal force no longer being a constant value, which can be changed by the fiber placement angle to alter the internal force distribution of the internal laminated structure. Therefore, the mechanical properties of the laminate structure can be optimized.

### 4.2. Stress Analysis of the Variable-Angle Laminate

**Figure 7:**
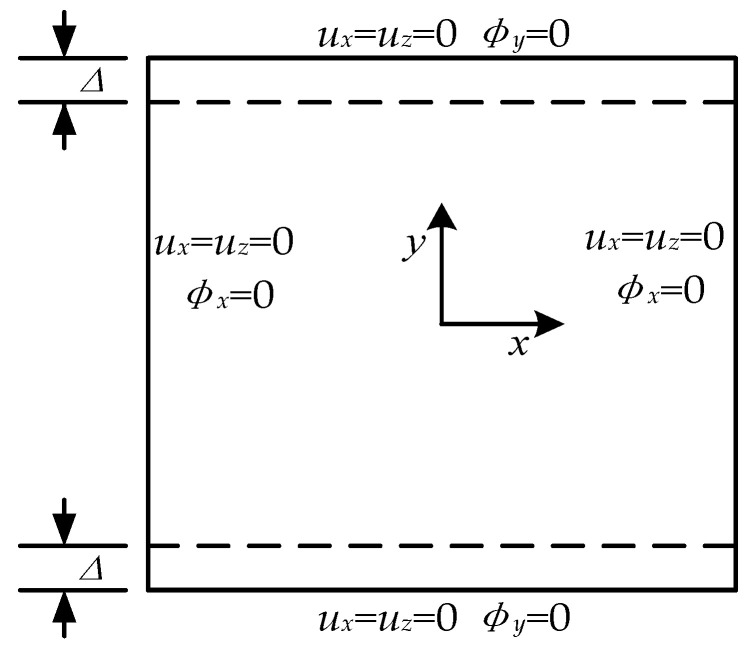
Static analysis model and boundary conditions.

In order to analyze the mechanical properties of variable-angle laminates, a laminate plate with a length of 150 mm is selected as the analytical model. As shown in Figure 7, the uniform Δ = 1 mm is exerted on the boundaries to compress the variable-angle laminates. The material of the analytical model is AS4/PEEK, and its elastic modulus and other related properties are summarized in Table 1 [30]. In this paper, the variable-angle laminate selected is [0± < 20 (β) 60) >]_2s_. The range of connection point parameter β is [0.1, 0.9]. The influence of β on the stress of variable-angle laminates can be observed.

**Figure 8:**
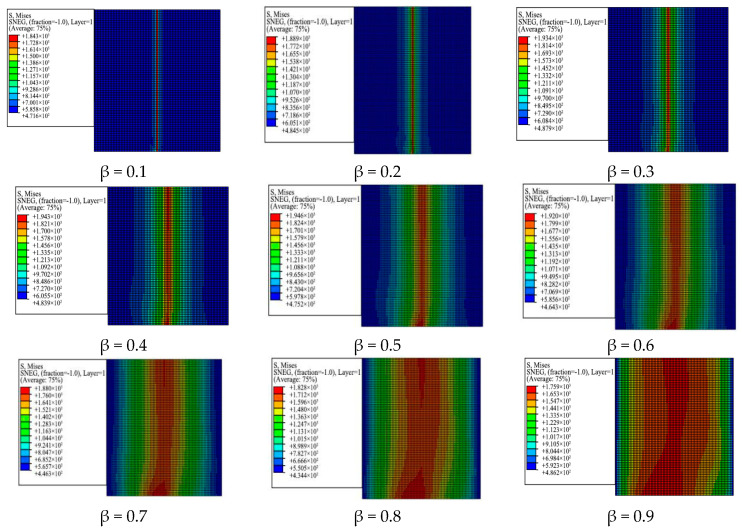
Equivalent stress distribution nephogram of variable-angle laminates of [0 ± < 20 (β) 60 >]_2s_.

Compared with traditional constant-angle laminates, the stress of variable-angle laminates also changes with the fiber angle due to the change of the arbitrary fiber placement angle. An equivalent stress distribution nephogram of variable-angle laminates of [0 ± < 20 (β) 60 >]_2s_ is illustrated in Figure 8. It can be found from the figure that the stress area of the variable-angle laminates changes with the increasing connection point parameter β. Since the left and right end faces are constrained by the boundary conditions, the stress in the middle area is the largest. The stress area gradually increases from the middle to the left and right ends, which indicates that the stress of variable-angle laminates is gentle, increasing the bearing capacity of the plate and avoiding stress concentration. From the stress area, it can be seen that the maximum stress area gradually expands from the small area in the middle to the two sides as the connection point parameter β changes. When the connection point parameter β is 0.9, the maximum stress area almost extends to the entire variable-angle laminate.

**Figure 9:**
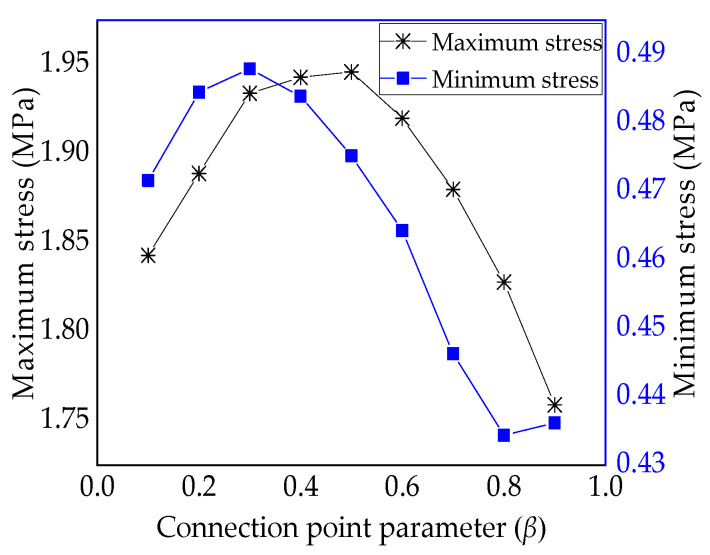
Maximum-minimum equivalent stress of variable-angle laminates of [0 ± < 20 (β) 60 >]_2s_ with connection point parameter β.

Table 2 presents the maximum-minimum equivalent stress of variable-angle laminates of [0 ± < 20 (β) 60 >]_2s_. According to the data in Table 2, the maximum and minimum stress curves are illustrated in Figure 9. The maximum stress value of the laminate increases first and then decreases as the connection point parameter β increases. When the parameter β is 0.5, it reaches the maximum value. The minimum stress value of the laminate increases initially and then decreases as the connection point parameter β increases. When the parameter β is 0.3, it reaches the maximum value. A high bearing capacity can be obtained by setting a reasonable connection point parameter β.

**Figure 10:**
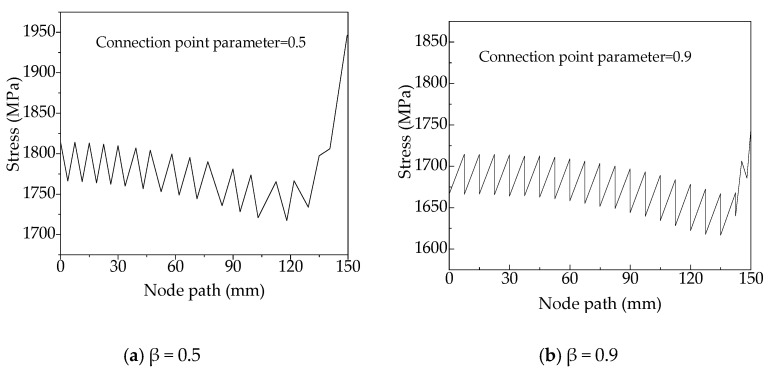
Stress distribution of variable-angle laminates of [0 ± < 20 (β) 60 >]_2s_ in the axial direction. (**a**) β = 0.5; (**b**) β = 0.9.

By comparing the maximum and minimum stresses of variable-angle laminates, it can be found that the maximum equivalent stress is one order of magnitude higher than the minimum equivalent stress. According to the change rule of the laminate equivalent stress, the equivalent stress values of variable-angle laminates [0 ± < 20 (0.5) 60 >]_2s_ and [0 ± < 20 (0.9) 60 >]_2s_ are selected as examples to observe the stress values of each node on the central axis of the laminate. Figure 10 shows that the stress distribution is along the axis of the variable-angle laminates of [0 ± < 20 (β) 60 >]_2s_: β = 0.5, β = 0.9. The upper end middle point of the laminate is the starting point along the axis, and the symmetrical lower end middle point is the end point of the axis (as shown by the arrow in the nephogram corresponding to β = 0.5 in (Figure 8)). It can be seen that the stress of variable-angle laminates decreases at first and then increases from the plate upper end when it reaches the maximum at the lower end. The equivalent stress occurs in a zigzag wave along the axis from the laminate upper end. This is due to the mechanical properties of the adjacent elements with different fiber placement angles. Therefore, the above-mentioned equivalent stress changing phenomenon occurs for variable-angle laminates.

### 4.3. Deformation Analysis of Variable-Angle Laminates

**Figure 11:**
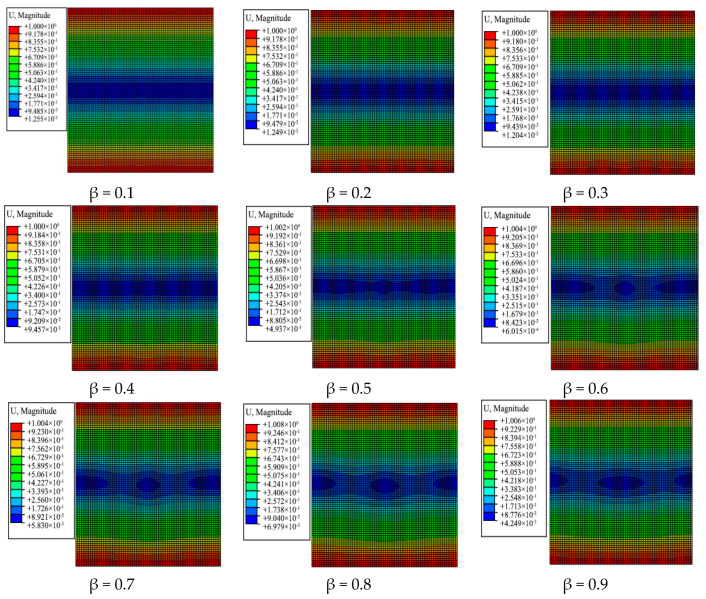
Deformation distribution nephogram of variable-angle laminates of [0 ± < 20 (β) 60 >]_2s_.

Compared with traditional constant-angle laminates, owing to arbitrary change of the fiber placement angle, the deformation in the laminate surface also changes with the fiber angle, and the deformation is not uniform. A deformation distribution nephogram of variable-angle laminates of [0 ± < 20 (β) 60 >]_2s_ is illustrated in Figure 11, where it is shown that that the deformation of the variable-angle laminate changes with the connection point parameter β. Since the upper and lower end faces are constrained by the boundary conditions, the maximum deformation occurs at the upper and lower ends. It gradually decreases toward the middle, and the deformation area has a symmetrical form. It can be seen from the deformation area that the initial regular rectangle gradually changes to an elliptical distribution and the maximum deformation area gradually decreases.

**Figure 12:**
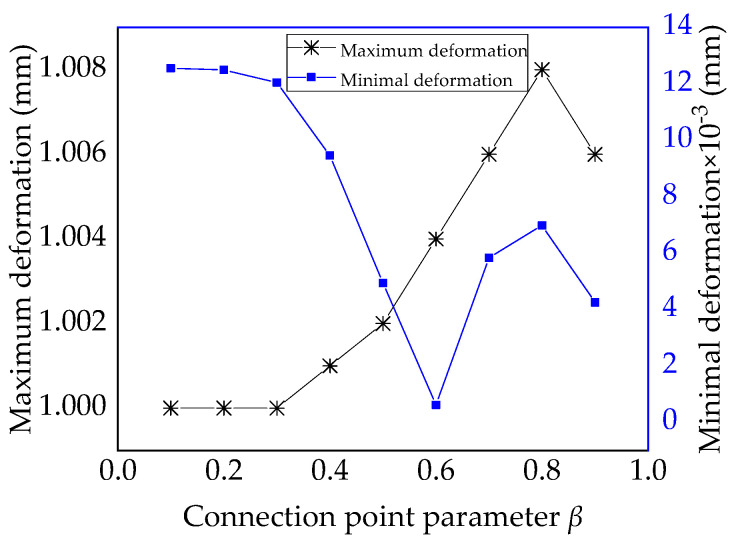
Maximum-minimum deformation of variable-angle laminates of [0 ± <20 (β) 60 >]_2s_ with connection point parameter β.

The maximum-minimum deformation of variable-angle laminates of [0 ± < 20 (β) 60 >]_2s_ is summarized in Table 3. According to the data in Table 3, the maximum and minimum deformation curves are plotted in Figure 12. The maximum deformation of the laminate increases initially and then decreases as the connection point parameter β increases. When the parameter β is 0.8, it reaches the maximum value. However, the minimum deformation of the laminate decreases at first and then increases as the connection point parameter β increases. When the parameter β is 0.6, it reaches the minimum value. For the quadratic Bezier curve method, the corresponding deformation can be changed by setting the connection point parameter β.

**Figure 13:**
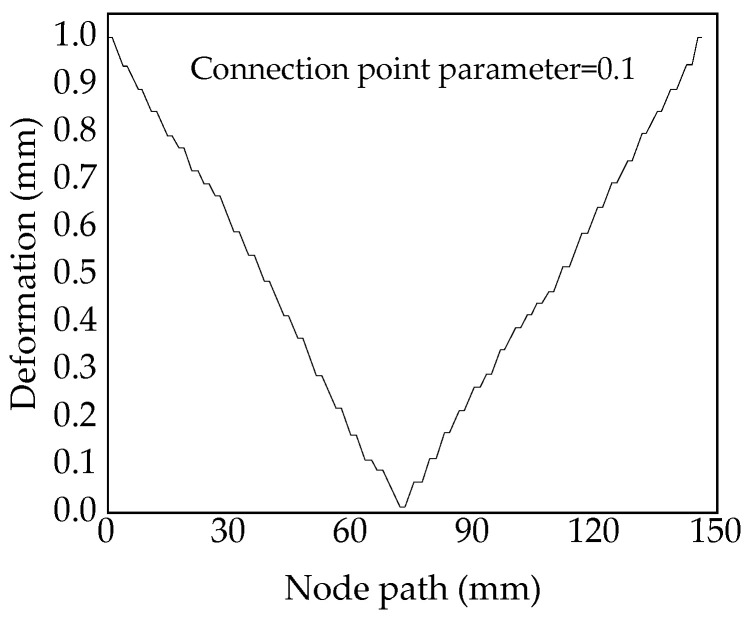
Deformation distribution of variable-angle laminates of [0 ± < 20 (0.1) 60 >]_2s_ in the axial direction.

According to the change rule of equivalent deformation in variable-angle laminates, the largest value among the minimum deformation of variable-angle laminates of [0 ± < 20 (0.1) 60 >] _2s_ is selected as an indicator to observe the deformation corresponding to each node on the central axis. The deformation distribution of variable-angle laminates of [0 ± < 20 (0.1) 60 >] _2s_ in the axial direction is shown in Figure 13. Among them, the middle point at the upper end of the laminate is the starting point, and the symmetrical middle point at the lower end is the end point (as shown by the arrow in the nephogram corresponding to β = 0.1 in Figure 11). It can be seen from Figure 13 that the deformation gradually decreases from the laminate upper end to the central position, reaching the minimum value, and then gradually increases. The curve has a symmetrical form. The trend of the deformation can also be seen in the nephogram.

### 4.4. Modal Analysis of Variable-Angle Laminates

**Figure 14:**
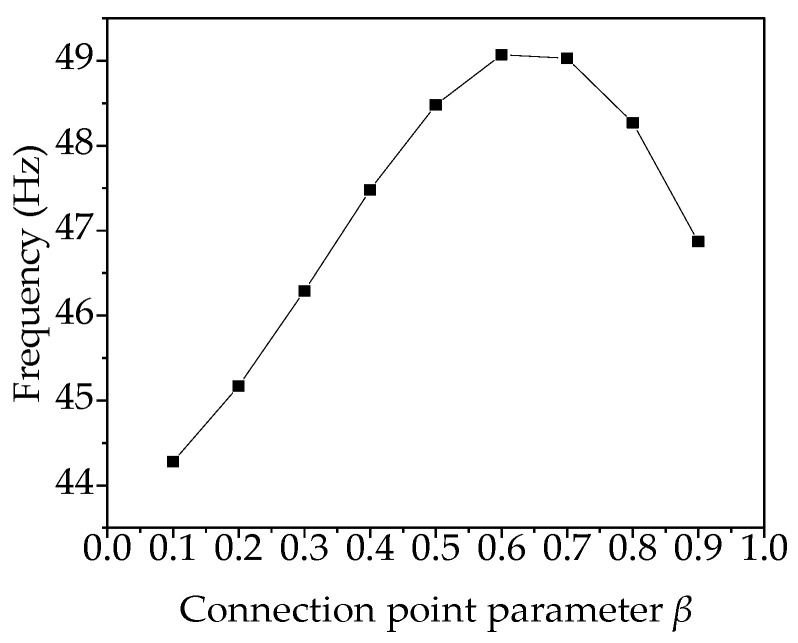
Relationship between the connection point parameter β and first-order frequency.

For variable-angle laminates, the vibration is generally analyzed with the first mode frequency as the main object. Taking the first-order frequency of the variable-angle laminates of [0 ± < 20 (β) 60 >]_2s_ as an example, the relationship between the first-order frequency and the connection point parameter β is shown in Figure 14. It can be seen from the figure that as the connection point parameter β changes, the first-order frequency increases firstly and then decreases with the increase of β. This indicates that the placement structure of variable-angle laminates of [0 ± <20 (β) 60 >]_2s_ changes with β, resulting in frequency change of variable-angle laminates.

**Figure 15:**
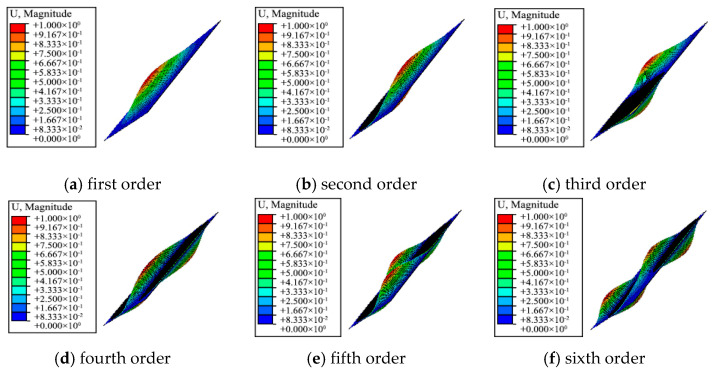
Six-order mode shape of variable-angle laminates of [0 ± < 20 (0.6) 60 >]_2s_.

Figure 15 illustrates the vibration shapes of the first six-order modes of the variable-angle laminates of [0 ± < 20 (0.6) 60 >]_2s_. The changes of the natural frequency and vibration shape are shown in Table 4. It can be seen from Figure 15 and Table 4 that the mode shape of the variable-angle laminated plate changes differently as each order frequency changes. Compared with constant-angle laminates, the frequency of fiber placement variable-angle laminates is more designable with the change of trajectory design parameters (connection point parameter β). The reasonable design parameters are optimized to adjust the frequency value of variable-angle laminates, which plays a certain guiding role in avoiding resonance.

## 5. Conclusions

In this paper, variable-angle laminates constructed by the quadratic Bezier curve method are taken as the research object. The correctness of the variable-angle laminate model established in this paper is verified by selecting variable-angle laminates corresponding to different connection point parameters β. Based on the constitutive equation theory of laminates, the stress and deformation of nine-group variable-angle laminates are analyzed. It can be seen from the results that the maximum stress area increases as the connection point parameter β increases. The maximum stress value of the laminate increases at first and then decreases as the parameter β increases. When the parameter β is 0.5, it reaches the maximum value. The minimum stress value of the laminate increases initially and then diminishes as the connection point parameter β increases. When the parameter β is 0.3, it reaches the maximum value. The stress of the variable-angle laminates decreases initially and then increases from the upper end of the plate and reaches the maximum value at the plate lower end. The equivalent stress occurs in a zigzag wave along the axis from the laminate upper end. The deformation of the variable-angle laminate is changed with the connection point parameter β. The maximum deformation of the laminated increases initially and then decreases as the parameter β increases. When the parameter β is 0.8, it reaches the maximum value. However, the minimum deformation of the laminate decreases at first and then increases as the connection point parameter β increases. When the parameter β is 0.6, it reaches the minimum value. It can be seen from the deformation area that the initial regular rectangle gradually changes to an elliptical distribution, and the maximum deformation area gradually decreases. The deformation gradually decreases from the upper and lower ends to the middle, and the deformation area has a symmetrical form. The initial regular rectangular area is gradually changed to an elliptical distribution and the area of maximum deformation gradually decreases.

Owing to the better designability of variable-angle laminates, the mechanical properties of such laminates are greatly improved compared with those of constant-angle laminates. The design and preparation of composite laminates conducted by using the mechanical properties analysis model of the variable-angle laminates proposed in this paper have higher value in engineering applications. Thermoplastic fiber placement technology is a multidisciplinary research field, and corresponding theoretical and experimental research is in continuous progress. In this paper, only a mechanical simulation of variable-angle laminates is presented, and future research work needs to be carried out in terms of placement experiments and mechanical property tests of laminates, in order to further verify the research and improve the application value of the results.

## Figures and Tables

**Table 1:** Material parameters of AS4/PEEK [30].

Parameter	Unit	Value
Elastic Modulus *E*_1_	GPa	139
Elastic Modulus *E*_2_	GPa	10.3
Shear modulus *G*_12_	GPa	5.2
Shear modulus *G*_13_	GPa	5.2
Shear modulus *G*_23_	GPa	3.96
Poisson’s ratio *μ*_12_		0.3

**Table 2:** Maximum-minimum equivalent stress of variable-angle laminates of [0 ± < 20 (β) 60 >]_2s_.

β	0.1	0.2	0.3	0.4	0.5	0.6	0.7	0.8	0.9
Maximum × 10^3^ (MPa)	1.843	1.889	1.934	1.943	1.946	1.92	1.88	1.828	1.759
Minimum × 10^2^ (MPa)	0.472	0.485	0.488	0.484	0.475	0.464	0.446	0.434	0.486

**Table 3:** Maximum-minimum deformation of variable-angle laminates of [0 ± < 20 (β) 60 >]_2s_.

*β*	0.1	0.2	0.3	0.4	0.5	0.6	0.7	0.8	0.9
Maximum (mm)	1	1	1	1.001	1.002	1.004	1.006	1.008	1.006
Minimum (mm × 10^−3^)	12.55	12.49	12.04	9.457	4.937	0.615	5.83	6.979	4.249

**Table 4:** Modal analysis of variable-angle laminates of [0 ± < 20 (0.6) 60 >]_2s_.

Orders	Frequency	Mode of Vibration
	(Hz)	
1	49.07	The plate center vibrates along the Z direction
2	83.53	The left and right sides of the plate center vibrate in the Z direction
3	107.77	The upper and lower sides of the plate center vibrate along the Z direction
4	133.38	The two diagonals of the plate vibrate in the Z direction
5	144.61	The center and diagonal of the plate vibrate in the Z direction
6	192.51	The upper, middle, and lower parts of the plate vibrate in the Z direction
